# Obituary: Timothy Meese (1964−2026)

**DOI:** 10.1177/03010066261470308

**Published:** 2026-08-07

**Authors:** Peter Thompson, Pascal Mamassian, Isabelle Mareschal, Annabelle Redfern, Frans A. J. Verstraten, Johan Wagemans

**Affiliations:** 1 8748 University of York, York, UK; 2 Laboratoire des systèmes perceptifs, Département d’études cognitives, École normale supérieure, PSL Université, CNRS, Paris, France; 3 Queen Mary University of London, London, UK; 4 1980 The University of Bristol, Bristol, UK; 5 University of Sydney, Sydney, NSW, Australia; 6 University of Leuven, Leuven, Belgium


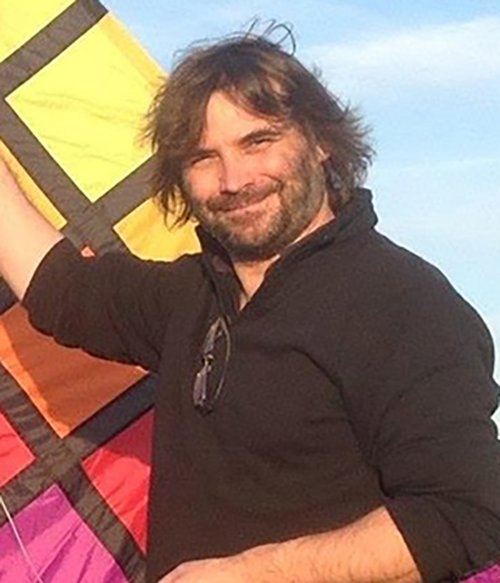


May 2026 was met with devastating news that quickly spread: our friend and co-editor-in-chief Timothy Meese, Tim to his friends, had died of a heart attack. He was only 61 years old with boundless energy and so much life yet to be experienced. At the time of his death, he was the most senior editor-in-chief of Perception and *i-*Perception. These journals were the projects where his heart was, where he served in several roles for two decades.

Tim joined the *Perception* managing team at the time the landscape of academic publishing was rapidly changing, and it was clear that *Perception* – then a very traditional journal – needed a fresh view. Tim's role was critical to the journal redefining itself over the next few years. That he was chosen for this role is a tribute to the outstanding range of his academic interests, the quality of his research and his personal qualities. Tim brought to the team an impressive range of interests: from the slope of the psychometric function, the application of our discipline to ‘real world’ situations, and in philosophical aspects of perception (see below). He was ideally placed to see the launch of *i*-Perception a year later and was very proud that last year's *i*-Perception Impact Factor was one of the highest among the vision and perception journals.

Tim was also happy in his role as an ambassador of our journals in Asia and Europe. He often spoke about how much he enjoyed the Asia Pacific Conference on Vision (APCV). And the same is true for the European Conference on Visual Perception (ECVP). He tried to be present every year; not only in his role as an academic and editor-in-chief, but also as the curator of Tom Troscianko's bequest after Tom's untimely death. At each ECVP conference, the Tom Troscianko Memorial Award (https://www.theava.net/awards/troscianko.php) is awarded to the person who travels to the host city in the most original way.

As mentioned, Tim was at the time of his passing the most senior editor-in-chief of our journals but had chosen to use this year as his sabbatical and, as a result, did not join us for our bi-annual face-to-face meeting at the Sage office in London. We missed his sharp wit during the traditional dinner the evening before our meeting. He was rejuvenating his research ideas and making new plans, especially to make his new initiative for our journal – *philosophy corner* – a success (see Meese et al., 2026). He was to introduce his latest ideas at the forthcoming ECVP in Bournemouth and was looking forward to catching up with his many friends there. We can only guess what his next ideas would have been, but we can ensure that Tim's *philosophy corner* will be part of his legacy. From a place far away, he might be following us and hoping we do the right thing.

We will meet in Bournemouth and drink a good pint, or a glass of fine wine as Tim would have preferred, in his memory.
